# Biodegradation: the best solution to the world problem of discarded polymers

**DOI:** 10.1186/s40643-024-00793-1

**Published:** 2024-08-07

**Authors:** Jun Wu, Jia Wang, Yicheng Zeng, Xinxiao Sun, Qipeng Yuan, Ling Liu, Xiaolin Shen

**Affiliations:** 1 State Key Laboratory of Chemical Resource Engineering, Beijing University of Chemical Technology, Beijing, 100029 China; 2 State Key Laboratory of Mycology, Institute of Microbiology, Chinese Academy of Sciences, Beijing, 100101 China

**Keywords:** Biodegradation, Polymers, Single-strain degradation, Multi-strain degradation, Enzyme engineering

## Abstract

The widespread use of polymers has made our lives increasingly convenient by offering a more convenient and dependable material. However, the challenge of efficiently decomposing these materials has resulted in a surge of polymer waste, posing environment and health risk. Currently, landfill and incineration treatment approaches have notable shortcomings, prompting a shift towards more eco-friendly and sustainable biodegradation approaches. Biodegradation primarily relies on microorganisms, with research focusing on both solitary bacterial strain and multi-strain communities for polymer biodegradation. Furthermore, directed evolution and rational design of enzyme have significantly contributed to the polymer biodegradation process. However, previous reviews often undervaluing the role of multi-strain communities. In this review, we assess the current state of these three significant fields of research, provide practical solutions to issues with polymer biodegradation, and outline potential future directions for the subject. Ultimately, biodegradation, whether facilitated by single bacteria, multi-strain communities, or engineered enzymes, now represents the most effective method for managing waste polymers.

**Graphical Abstract:**

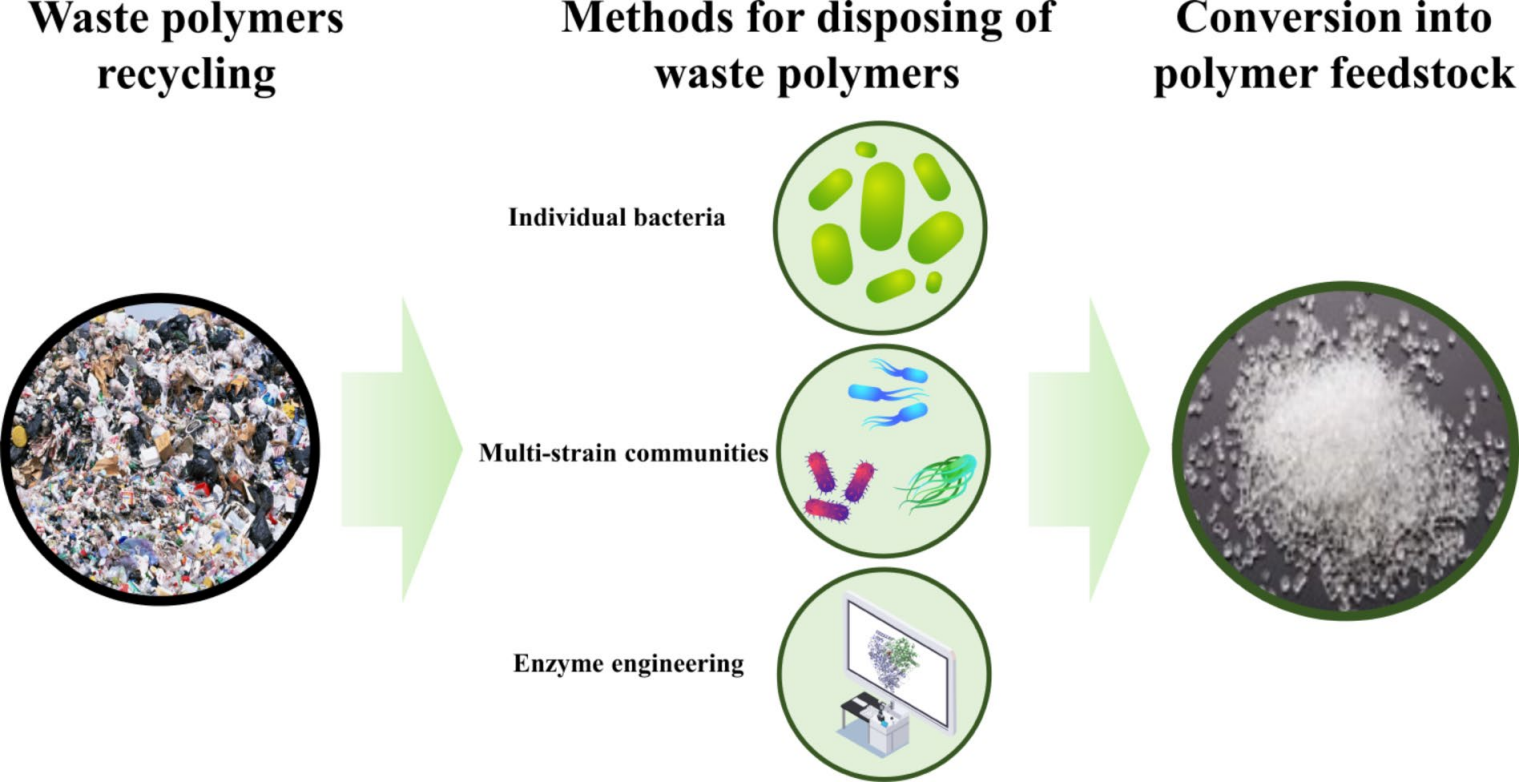

**Supplementary Information:**

The online version contains supplementary material available at 10.1186/s40643-024-00793-1.

## Introduction

Polymers, comprise polymerizing small-molecule connecting monomers, encompass a wide range of materials such as rubber, plastic, polyester. Over the past few decades, polymers have played a pivotal role in everyday life and industrial production, becoming an integral part in various facets of our lives. The demand for polymers has surged, leading to annual production growth. In 2022, production reached 400.3 million tons, which is a significant increase of 6.3 million tons compared with the previous year (Europe 2023). The increasing production comes with a significant surge in polymer waste. By 2050, 12 billion metric tons of waste polymers will be generated and released into the environment (Amobonye et al. 2021). This influx of polymer waste poses severe threats to ecosystem. It can contaminate water supplies, accumulate in the human body, entangle marine life, and adversely affect navigation (Ahari and Soufiani 2021; Béraud et al. 2022; Brasika et al. 2023; Lusher et al. 2018; Schmaltz et al. 2020).

**Fig. 1:**
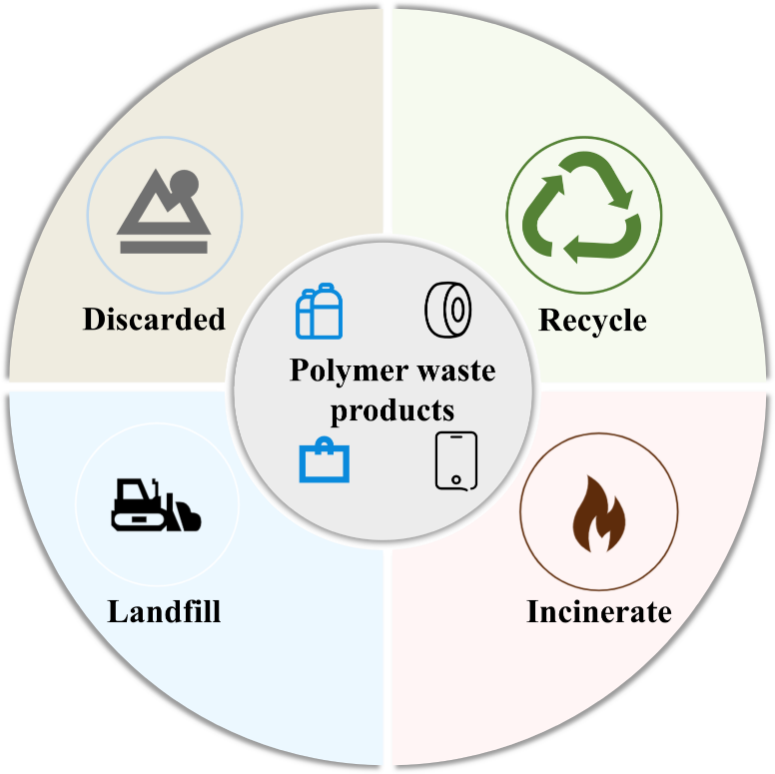
The main destinations of the polymers

The disposal of waste polymers typically involves methods such as landfill, which exerts a significant demand on land resources. As these waste polymers gradually degrade within landfills, microplastics and monomers continue to leach out, causing severe contamination of the surrounding soil and groundwater (Wang et al. 2019a; Yan et al. 2023). The endurance of polymers exacerbates this damage, leading to long-lasting environmental consequences. Another prevalent disposal method is incineration, a process that releases hazardous chemicals into the atmosphere, including carbon dioxide, sulfur dioxide, and dioxins (Zhao et al. 2020). In addition, incineration produces a large amount of hazardous and poisonous dust and slag compounds, which pose a significant threat to the environment (Duval 2014; Wang et al. 2019a; Yan et al. 2023). The main destinations of the polymers now are shown in Fig. 1.

**Fig. 2:**
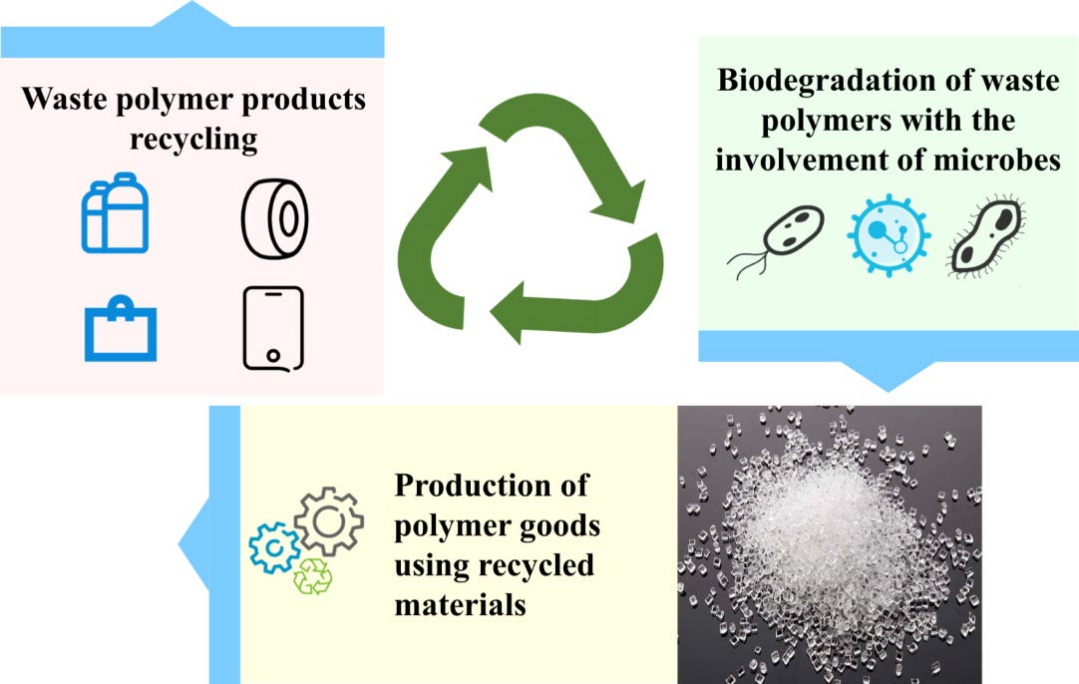
The schematic diagram of the recycling

Previous researches have shown that biodegradation stands out as a sustainable and ecologically friendly process for handling waste polymers. Utilizing biological enzymes and microbial strains, biodegradation converts waste polymers into smaller molecules or even monomers. Biodegradation, when contrasted with traditional landfill methods, has the ability to preserve valuable land resources by reducing solid volume by up to 80% (Mostafa et al. 2018; Rai et al. 2021). Moreover, biodegradation exhibits a superior capacity for degradation. The rapid decomposition of the polyester polymer polyethylene terephthalate (PET) exemplifies the high efficiency of biodegradation. Contrary to the lengthy natural deterioration process that takes place in landfills over several decades, biodegradation can rapidly disintegrate PET within a few days (DelRe et al. 2021). Furthermore, biodegradation plays a crucial role in reducing environmental pollutants. It can eliminate harmful gases, such as dioxins, and reduce greenhouse gas emissions by 60% when compared to incineration (Rajendran and Han 2022). Beyond waste reduction, biodegradable polymer monomers generated through this process can contribute to recycling efforts and diminish the reliance on fossil fuels. These monomers have the capability to polymerize into new macromolecular polymers, boosting recovery rates by 40% (Rajendran and Han 2022). The establishment of a “recycle-generate-reuse” green cycle is made possible by employing biodegradable treatments in polymer recycling, as depicted in Fig. 2. In conclusion, the most effective and environmentally sound approach to handle waste polymers is through biodegradation.

Various microorganisms have undergone trails for polymer degradation over the past few decades. Despite efforts to identify more effective strains through screening, most individual wild strains exhibit limited degradation capabilities. Aiming to achieve efficient biodegradation of diverse polymers, multi-strain communities have been proposed as an alternative to individual strains, as communities offer greater resilience to deterioration and higher efficiency. Furthermore, there is growing interest in enhancing the ability of strains to degrade polymers, which can be achieved by utilizing enzyme engineering to modify their catalytic enzymes and increase their catalytic efficiency. Compared with the previous review (Kotova et al. 2021; Pathak and Navneet 2017), this review offers a comprehensive introduction to the three key areas of biodegradation, and provides a rounded analysis of all categories of polymers. The analysis traces the progression and transformation of polymer degradation, starting from the examination of wild individual strains to communities and then to genetically modified strains. The factors driving the advancement and evolution of this process are elucidated, offering a concise overview of the present state of the field. The subsequent text examines the constraints of biodegradation and presents viable solutions that are derived from the latest advancements in the field. In the final phases, the text provides a perspective on the anticipated advancements and potential discoveries in the field of biodegradation.

## Biodegradation based on a single strain

**Fig. 3:**
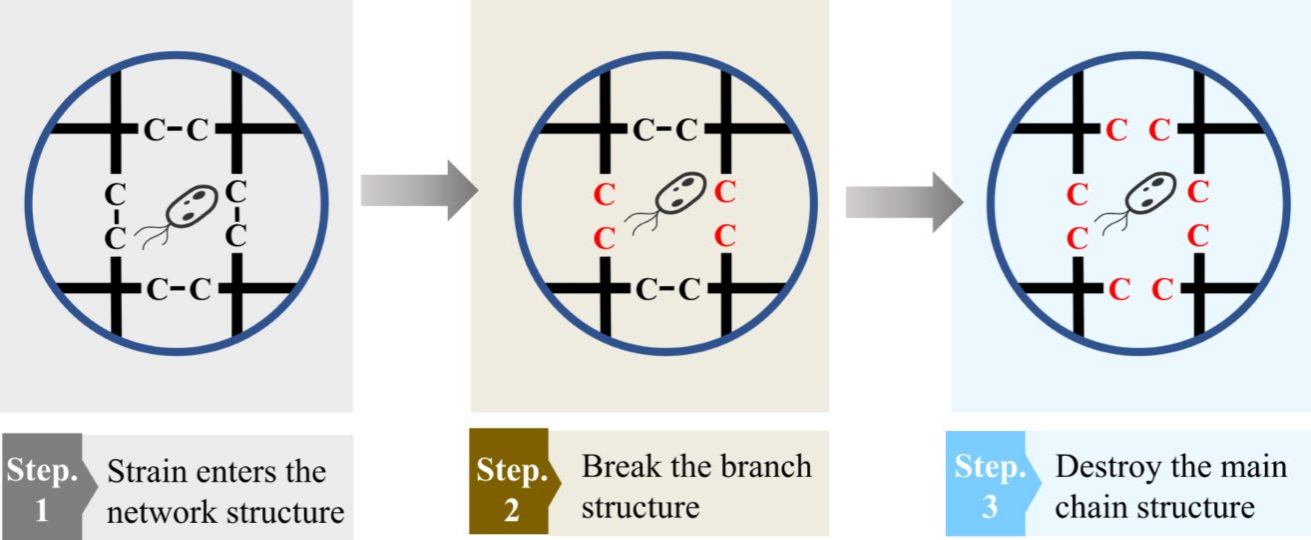
The process of polymer degradation by a single strain

**Fig. 4:**
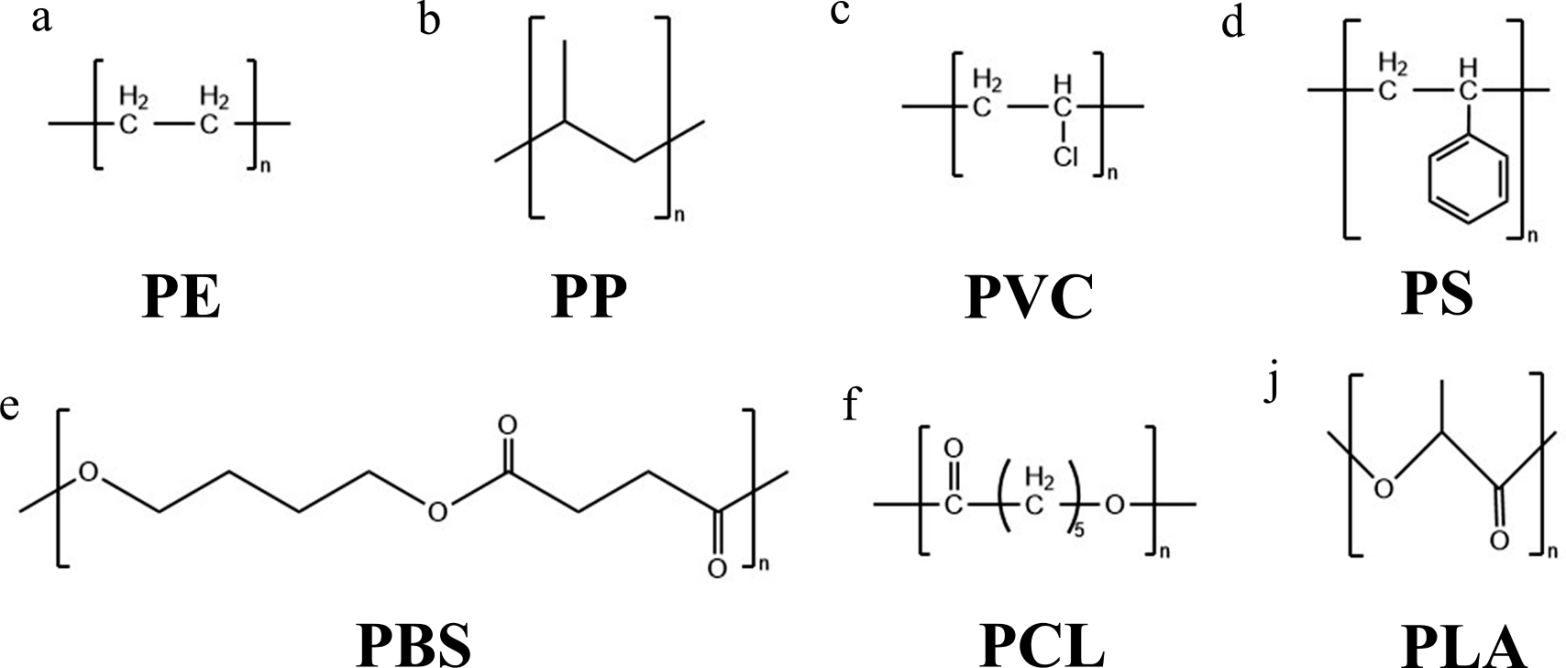
The chemical structures of the polymers mentioned in the text

Biodegradation reactions vary among different polymers, for example, *Pseudomonas citronellolis* was able to degrade PVC but not PP (Giacomucci et al. 2019). Therefore, we categorize biodegradation based on the specific polymer type, such as rubber, plastics, and polyester. Rubber is a naturally occurring polymer composed of *cis*-1,4-polyisoprene (Andler 2020). However, natural rubber has lower stability and abrasion resistance. Typically, natural rubber undergoes a process called vulcanization to create vulcanized rubber (Wiśniewska et al. 2022). This process leads to rubber with enhanced properties, including greater stability, abrasion resistance, and lower degradability. Plastics are synthetic materials created by combining or condensing monomers, such as polyethylene (PE), polypropylene (PP), polyvinyl chloride (PVC), and polystyrene (PS), which are widely utilized. The primary distinction between plastics and rubber lies in their deformation behavior. Plastic deforms plastically, while rubber deforms elastically (Zhigang and Kuangdi 2022). Polyesters are polymers created through esterification processes. The cross-linking mechanism, also known as the esterification reaction, is reversible and can probably be readily undone through hydrolysis (Satti and Shah 2020). This property renders polyesters biodegradable. “Additive polymers” refers to a specific category of polymers that are improved for degradability by the addition of compatibilizers. The process of polymer degradation by a single strain is shown in Figs. 3 and 4 depicts the chemical structures of the majority of the polymers mentioned in the text.

## The degradation process of rubber

Rubber is a natural polymer and was one of the earliest polymers employed by mankind. It is extensively used in the production of several goods, including mattresses, tyres, gloves, and pillows (Prasopdee and Smitthipong 2020). Natural rubber (NR) may be broken down by a variety of naturally occurring bacteria. The original researchers investigated how well a variety of wild bacteria might degrade rubber using randomized trials. *Rhodococcus pyridinivorans* F5 exhibited the highest efficiency in degrading natural rubber, resulting in an 18% decrease in rubber weight over a 30 days period (Nawong et al. 2018). Subsequent researchers systematically examined and separated wild strains by using rubber as the sole carbon source, rather than making randomized trials (Altenhoff et al. 2021; Basik et al. 2022; Sarkar et al. 2021; Schmitt et al. 2019). By employing this method, they successfully isolated 50 species of bacteria capable of degrading rubber from natural environments. Additionally, another 46 species of rubber-degrading bacteria were identified through screening multiple strain collection centers (Schmitt et al. 2019). After incubating these selected strains in medium with NR as a carbon source for 30 days, the weight of NR was decreased by 10–30% and the average molecular weight of polymer decreased from 640 kDa to 25 kDa (Schmitt et al. 2019). All of the isolated strains were identified as *Actinomyces* spp. They were easy to distinguish during the initial screening procedure because they could produce hyphae and create transparent areas when decomposing rubber on agar plates with NR. Screening bacteria for rubber degradation by this screening process can be challenging when the identifying bacteria exhibit no obvious indications of reaction. *Actinomycetes*, such as *Nocardia* and *Mycobacterium*, as well as non-actinomycetes like *Corynebacterium*, do not form a transparent areas when degrading rubber. This makes it more difficult to screen for these strains (Basik et al. 2021; Prakash et al. 2024). Previously, it was believed that only Gram-positive bacteria were capable of degrading natural rubber since all mentioned rubber-degrading strains were identified as such. These strains that degrade rubber contain the *lcp* gene or its homologous sequence, which codes for the latex cleavage protein (LCP). LCP is recognized as a crucial enzyme for degrading natural rubber, as the insertion of the *lcp* gene enables *Escherichia coli* to degrade natural rubber (Basik et al. 2022). Nevertheless, Sharma’s discovery of *Steroidobacter cummioxidans* strain 35Y challenged the perspective that only Gram-positive bacteria have the ability to degrade rubber. This strain was demonstrated to be the most effective among all known strains. Within seven days, this particular strain led to a 60% decrease in NR (Cui et al. 2023; Sharma et al. 2018). Imai et al. identified and separated three additional gram-negative bacteria capable of rubber degradation: *Streptomyces* sp. LCIC4, *Actinomyces* sp. OR16, and *Methylating bacteria* sp. NS21. LCIC4 exhibited the highest rubber degradation capability, degrading 70% of NR in just 50 days. This degradation resulted in an average reduction in molecular weight of rubber from 400 to 23 kDa (Imai et al. 2011). The identification of rubber oxygenase (RoxA), a key enzyme required for the degradation of NR in gram-negative bacteria, was first reported in *Xanthomonas* 35Y. The enzymes RoxA and LCP play a crucial role in the degradation of NR by breaking the double bond between rubber monomers (cis-1,4-polyisoprene) through oxidative cleavage (Suzuki et al. 2022).

Vulcanization is a commonly used process to enhance the stability and optimize the properties of natural rubber. Nevertheless, microbial strains capable of degrading NR are unable to directly degrade vulcanized rubber due to their inability to break the C-S or S-S bonds present in vulcanized rubber. In other words, these strains lack the capacity for desulfurization. These strains can only participate in the degradation of vulcanized rubber after it has been desulfurized. Consequently, the initial process of degrading vulcanized rubber can be split into two distinct steps: desulphurization and degradation. The elimination of sulfur from vulcanized rubber is mostly accomplished by the use of various species of *Thiobacillus* bacteria. For instance, the content of sulfur in vulcanized rubber decreases by 30% after undergoing a 30 days treatment with *Thiobacteria* (Calabrese et al. 2021). Subsequently, it is necessary to introduce microorganisms that can degrade rubber in order to dismantle the primary chain structure. This complicates the degradation process of vulcanized rubber. To simplify and optimize the biodegradation of vulcanized rubber, it would be advantageous to have a single strain capable of achieving both desulfurization and degradation. Further research revealed that certain microorganisms have the ability to carry out desulfurization and degradation processes simultaneously. This means that a singular species of microbe can decompose vulcanized rubber. Aboelkheir et al. discovered that *Bacillus subtilis*, *Pseudomonas aeruginosa*, and *Streptomyces* possess the capacity to degrade vulcanized rubber. The utilization of these three strains on vulcanized rubber resulted in a decrease in cross-linking within the vulcanized structure by 17.2%, 10.7%, and 43.4%, and a reduction in the cleavage of C-C bonds by 16.1%, 16.8%, and 18.1%, respectively, as compared to the control. The data suggest that the desulfurization reaction and rubber degradation occurred simultaneously, which means that the vulcanized rubber was directly degraded (Aboelkheir et al. 2019). *Ceriporiopsis subvermispora*, a type of White-rot basidiomycetes, demonstrates superior efficiency in both desulfurizing and degradation of the main structure of vulcanized rubber compared to other organisms. Exposure of vulcanized rubber to *Ceriporiopsis subvermispora* for 200 days led to a 29% decrease in sulfur content and a 69% decrease in the frequency of S-C bonds. In contrast, the control group showed no degradation of S-C bonds or removal of sulfur (Chen et al. 2020). The main microorganisms capable of degrading rubber and their degradation capabilities are summarized in Table 1.

**Table 1:** Summary of strains with the ability to degrade rubber

Species	Rubber degradation capability	Ref.
*R. pyridinivorans* strain F5	In 30 days, rubber can lose up to 18% of its weight.	Nawong et al. 2018
*Xanthomonas* sp. strain 35Y	After one week of treatment, NR lost around 60% of its weight.	Sharma et al. 2018
*Streptomyces* sp. strain LCIC4	According to GPC research, the rubber’s average molecular weight dramatically dropped after 30 days and nearly completely disintegrated after 50 days.	Imai et al. 2011
*Actinoplanes* sp. strain OR16	According to GPC research, the rubber’s average molecular weight dropped after 30 days.	Imai et al. 2011
*Methylibium* sp. strain NS21	According to GPC research, the rubber’s average molecular weight slightly dropped after 30 days.	Imai et al. 2011
*Thiobacillus perometabolis*	Up to 30% less sulfur was present after the 30 days microbial treatment than in the control group.	Calabrese et al. 2021
*Bacillus subtilis* ATCC 6633	The loss of crosslinking is 17.2%, and the loss of contact angle with water is 14.1%, causing a carbon loss of 16.1%.	Aboelkheir et al. 2019
*P. aeruginosa* ATCC 9027	The loss of crosslinking is 10.7%, and the loss of contact angle with water is 12.9%, causing a carbon loss of 16.8%.	Aboelkheir et al. 2019
*Streptomyces*	The loss of crosslinking is 43.4%, and the loss of contact angle with water is 15.7%, causing a carbon loss of 18.1%.	Aboelkheir et al. 2019
*C. subvermispora*	The fungus decreased the total sulfur content of the rubber by 29% in 200 days, and S − C bonds decreased by 69%.	Chen et al. 2020

## Degradation of the plastic family

Plastic is a broad term that encompasses various polymers, such as polyethylene (PE), polypropylene (PP), polyvinyl chloride (PVC), and polystyrene (PS). These materials have taken the place of wood, glass, and metal in several applications. Plastics, renowned for their durability, were once believed to be non-biodegradable unless subjected to certain treatments. However, the pretreatment of plastics is a complicated and energy-intensive procedure, so it is necessary to investigate strains that can directly degrade polymers.

PE is a highly prevalent thermoplastic material. Early studies have indicated that certain types of bacteria, such as *Bacillus*, *Rhodococcus*, and *Pseudomonas*, as well as fungi like *Aspergillus* and *Fusarium*, are capable of degrading polyethylene when exposed to ultraviolet (UV) radiation or heat treatment (Gómez-Méndez et al. 2021; Soong et al. 2023; Sun et al. 2022a). Recent studies have identified several microorganisms capable of degrading PE without the need for any special treatment, including *Pseudomonas putida* IRN22, *Acinetobacter peddler* IRN19, *Micrococcus luteus* IRN20, *Pseudomonas aeruginosa* PAO1, *Pseudomonas aeruginosa* ATCC, *Pseudomonas putida*, *Pseudomonas syringae*, *Pseudomonas* sp. E4, *Comamonas*, *Delftia*, and *Maltophilia* (Meyer Cifuentes et al. 2023; Montazer et al. 2019; Peixoto et al. 2017; Wei et al. 2022). Nevertheless, their ability to degrade PE is limited, resulting in less than a 1% loss in PE weight. This presents a challenge for the efficient polymer degradation and recycling. In the following studies, four strains, were isolated from the marine environment: *Cobetia* sp. H-237, *Halomonas* sp. H-255, *Exigobacterium* sp. H-256, and *Alcanivorax* sp. H-265. All of these bacteria exhibited the ability to degrade substances. Among these strains, H-255 had the most potent ability, resulting in a weight loss of 1.7% in PE after 90 days of treatment (Khandare et al. 2021). A separate team of scientists has discovered three additional bacteria, including *Kocuria palustris* M16, *Bacillus pumilus* M27, and *Bacillus subtilis* H1584, which exhibit superior degrading capabilities in the pelagic waters of the Indian Ocean. The weight loss of PE after 30 days of treatment with these three strains was 1.5%, 1.7%, and 1.8%, respectively, while the crystallinity dropped by 10.3%, 8.6%, and 4.6%, respectively (Harshvardhan and Jha 2013). Up to now, *Bacillus subtilis* H1584 has been proven to be the most effective strain for degrading PE directly. While these strains had limited degradation capacity, with none of them causing PE weight losses above 2%, this finding is significant since it challenges the conventional belief that PE is not inherently biodegradable.

Similar to PE, PP is also a widely used plastic, but these has been little research on its direct degradation. Most research has employed pre-treatment techniques, including γ-irradiation, UV irradiation, and heat treatment, followed by deterioration. For example, *Bacillus* is capable of degrading UV-treated PP (Devi et al. 2023). Only three strains of *Pseudomonas* spp., *Vibrio* sp., and *Alcaligenes* spp. have been shown to degrade PP without any prior treatment (Kelly et al. 2021). However, the degradation efficiency is minimal, with only 4% degradation observed after 40 days treatment.

There have been few attempts to biodegrade PVC due to its high stability. Only a small number of microorganisms have demonstrated the capability to degrade PVC (Giacomucci et al. 2019; Shilpa et al. 2022). *White-rot basidiomycetes* degrade low-molecular-weight PVC in circumstances under limited nutrients conditions (Chow et al. 2023). Both *Pseudomonas citronellolis* and *Bacillus flexus* demonstrate depolymerization activity on PVC films (Giacomucci et al. 2019). The combination of *Ascomycetes* and *Chaetomium globosum* (ATCC 16021) has demonstrated the capacity to degrade PVC (Vivi et al. 2019). Although these strains exhibited the capacity to degrade PVC, the extent of degradation is limited. Nevertheless, the co-cultivation of *Pseudomonas citronellolis* and *Bacillus flexus* on a PVC sheet exhibited robust degrading activity. After a 45 days treatment period, the average molecular weight of the PVC was decreased by 10%, accompanied by a weight loss of 19% approximately (Giacomucci et al. 2019). This is the sole example demonstrating a greater effect of biodegradation treatment on PVC.

PS is a thermoplastic material that has exceptional optical and chemical properties. It is worth noting that this type of plastic is highly biodegradable and can undergo degradation without any prior treatment. *Geobacillus stearothermophilus* FAFUA011, *Bacillus cereus*, and *Bacillus gottheilii cereus* are capable of thriving on PS, which was the only available carbon source (Xing et al. 2021). Treatment of PS with actinomycetes (*Rhodococcus ruber* sp. C208) resulted in a weight reduction of 0.8% over 8 weeks (Sun et al. 2022b). After undergoing a 56 days treatment with *Geobacillus stearothermophilus* FAFUA011, PS exhibited a 4.2% decrease in mass and a decrease in the average molecular weight ranging from 17.4 to 18.2% (Xing et al. 2021). The weight of PS decreased 5.8% and 7.4% after being treated with *Bacillus cereus* and *Bacillus gottheilii* for 40 days, respectively (Auta et al. 2017). To a certain extent, PS is one of the most easily degradable plastics.

In short, the majority of plastics are not biodegradable unless they are pre-treated. Additionally, the requirement for pretreatment can increase the intricacies and costs of degradation process. Plastics are typically stable and not easily biodegradable. Recent investigations have challenged the conventional belief that plastics are not biodegradable by nature. The main microorganisms capable of degrading plastics and their degradation capabilities are summarized in Table 2. Nevertheless, the direct biodegradation process exhibits limited efficiency, as the majority of plastics undergo direct biodegradation, resulting in a mass loss of 10% or less. Consequently, further in-depth studies are necessary.

**Table 2:** Summary of strains with the ability to degrade plastics

Species	Plastics degradation capability	Ref.
*K. palustris* M16	10.3% decrease in crystallinity after 30 days of incubation for M16.	Harshvardhan and Jha 2013
*B. pumilus* M27	8.6% decrease in crystallinity after 30 days of incubation for M27.	Harshvardhan and Jha 2013
*B. subtilis* H1584	4.6% decrease in crystallinity after 30 days of incubation for H1584.	Harshvardhan and Jha 2013
*Cobetia* sp. H-237	The dry weight loss of LDPE films after 90 days of incubation was 1.4%, The carbon remineralization study showed that the decomposition of LDPE film into CO_2_ on 15 days was 34.4 mg CO_2_ g^− 1^.	Khandare et al. 2021
*Halomonas* sp. H-237	The dry weight loss of LDPE films after 90 days of incubation was 1.7%, The carbon remineralization study showed that the decomposition of LDPE film into CO_2_ on 15 days was 26.1 mg CO_2_ g^− 1^.	Khandare et al. 2021
*Exigobacterium* sp. H-256	The dry weight loss of LDPE films after 90 days of incubation was 1.3%, The carbon remineralization study showed that the decomposition of LDPE film into CO_2_ on 15 days was 33.7 mg CO_2_ g^− 1^	Khandare et al. 2021
*Alcanivorax* sp. H-265	The dry weight loss of LDPE films after 90 days of incubation was 1.0%, The carbon remineralization study showed that the decomposition of LDPE film into CO_2_ on 15 days was 33.6 mg CO_2_ g^− 1^.	Khandare et al. 2021
*Pseudomonas* sp.	After 40 days of incubation, the polypropylene film sample had only 4% degradation.	Kelly et al. 2021
*Vibrio* sp.	After 40 days of incubation, the polypropylene film sample had only 4% degradation.	Kelly et al. 2021
*Alcaligenes* sp.	After 40 days of incubation, the polypropylene film sample had only 4% degradation.	Kelly et al. 2021
*P. citronellolis* and *B. flexus*	The average molecular weight of PVC is reduced by 10%, and the weight loss of up to about 19%	Giacomucci et al. 2019
*R. ruber* C208	With the polymer, average molecular weight and average molecular number decreased by 20% and 15%, respectively.	Sun et al. 2022b; Tao et al. 2023
*G. stearothermophilus* FAFU011	During 56 days of degradation, FAFU0011 caused a total mass loss of PS of 4.2% and a decrease in molecular weight of 17.4-18.2%.	Xing et al. 2021
*B. cereus*	After 40 days, the percentage weight loss of PE, PET, and PS by *B. cereus* was 1.6%, 6.6%, and 7.4%, respectively.	Auta et al. 2017
*B. gottheilii*	Recorded a percentage weight loss of 6.6%, 3.0%, 3.6%, and 5.8% for PE, PET, PP, and PS microplastics, respectively.	Auta et al. 2017

## Degradation of the polyester family

Compared with plastics, polyesters can undergo biodegradation through hydrolysis reactions, as they are cross-linked through esterification reaction. Polyesters, including polybutylene succinate (PBS), polybutylene succinate butadiene styrene (PBSA), polybutylene succinate butadiene terephthalate (PBAT), polycaprolactone (PCL), and polylactic acid (PLA) are environmentally friendly and biodegradable (Jiang et al. 2024; Satti and Shah 2020).

PBS is a type of thermoplastic polyester with exceptional processing and mechanical properties. PBS is classified as a highly degradable substance because it is formed as a polyester (Zhang et al. 2019). PBS, PBSA, and PBAT exhibit comparable degradation characteristics due to their esterification through a common group called butylene glycol ester. According to reports, lipase and cutinase enzymes released by bacteria can catalyze the hydrolysis of ester bonds, leading to the degradation of polyesters (Lin et al. 2019; Shi et al. 2019). Nevertheless, degradation by cutinase results in degradation byproducts that have a diameter three times greater than that of lipase. Furthermore, the PBS degraded by cutinase does not exhibit any alterations in crystallinity, whereas the lipase-degraded PBS demonstrates a gradual reduction in crystallinity over time (Shi et al. 2019). Therefore, the degradation of PBS is typically addressed by utilizing microorganisms capable of excreting lipase or by directly application of lipase. For instance, viscous lipase F, the enzyme was found in *Rhizopus niveus*, is capable of fully degrading PBS in a span of 17 days at a temperature of 37 °C and a pH level of 7.0. The Asahi lipase from *Chromobacterium viscosum* can completely degrade PBSA in only 4 days (Arunrattanamook et al. 2023). Also, *Rhizopus niveus*, *Alcaligenes* sp., and *Rhizopus oryzae* have the ability to fully degrade PBSA within 6, 11, and 22 days, respectively (Arunrattanamook et al. 2023).

PCL possesses exceptional characteristics, such as biocompatibility, favorable biodegradability, and compatibility with various materials. It is synthesized through the ring-opening polymerization of the ε-caprolactone monomer. Therefore, PCL is extensively utilized in the manufacturing of drug carriers, plasticizers, and biodegradable polymers. Various microbial strains can efficiently degrade PCL with high degradation efficiency. For example, treatment of PCL with *Chaetomium globosum* ATCC 16,021 for 28 days resulted in the formation of visible micropores and cracks, which caused a significant mass reduction of 75% (Vivi et al. 2019). Ahmed Nawaz obtained *Brevundimonas* sp. MRL-AN1 from the soil, which efficiently decomposed over 80% of the PCL within 10 days. The enzyme excreted by this strain exhibited stability throughout a broad spectrum of temperatures (20–45 °C) and pH levels (5–9), as well as in the presence of diverse metal ions, surfactants, and organic solvents. These characteristics render it appropriate for the decomposition of polyesters in intricate circumstances (Nawaz et al. 2015). Furthermore, *Brevundimonas* sp. MRL-AN1 has shown significant efficacy against various additional polyesters. Microorganisms capable of degrading polyesters can be found in various circumstances, including extreme conditions such as Antarctica. These microorganisms are not limited to typical soils. Aneta isolated 161 bacterial and 38 fungal strains from soil samples collected in Antarctica, all of which possess the ability to biodegrade polyesters. Over 92% of the bacteria and 77% of the fungus exhibited a high degrading activity, resulting in the etching and notching of PBSA, PBS, and PCL. *Sclerotinia* sp. B11IV and *Fusarium* sp. B3’M exhibited significant biodegradation activity, degrading 49.7% of PBSA and 33.7% of PCL, and 46.0% of PBSA and 49.7% of PCL, respectively. These two strains had the lowest optimal temperature requirement of 20 °C compared to all other strains that degrade polyester (Urbanek et al. 2021). All of these strains exhibited considerable PCL degradation efficiency, but none of them reached the highest level. The most effective strain for degrading PCL is *Penicillium oxalicum* strain DSYD05-1, which was created by Fan using UV irradiation of *Penicillium oxalicum*. This strain resulted in up to 80% weight loss after treating PCL for 6 days (Khatua et al. 2024).

PLA is a completely biodegradable polyester produced by the polymerization of lactic acid from the conversion of a renewable resource such as starch. The monomeric lactic acid in PLA can be directly metabolized by microorganisms. Research indicates that *Rhodopseudomonas palustris* exhibits remarkable degradation efficiency of PLA, capable of degrading approximately 40% of it within 36 h (Hajighasemi et al. 2016). The efficient degradation of PLA by the strain was principally facilitated by the release of its hydrolase RPA1511. Further investigations revealed and recognized conservative sites (Tyr139, Tyr213, Arg259, and Thr46) of the RPA1511 hydrolase. Modifying amino acids at specific sites such as His114, Trp219, and Ala273 was found to enhance the reaction’s effectiveness, whereas modification of amino acids at Arg244 hindered the reaction (Wang et al. 2019b). In short, various modifications to the enzyme all had the capacity to impact the degraded effectiveness of PLA by the strain.

In conclusion, all polyesters exhibit exceptional biodegradability and can be naturally degraded in environmental conditions. Nevertheless, the process of degradation can be expedited, and the efficiency of waste treatment can be enhanced by conducting a screening to identify the dominant strains. Proteins extracted from bacteria with the ability to degrade polyesters are summarized in Table 3.

**Table 3:** Summary of proteins derived from bacteria with the ability to degrade polyesters

Protein name	Source	Polyesters degradation capability	Ref.
Lipase F	*R. niveus*	Degrade PBS within 17 days at 37 °C and pH 7.0.	Arunrattanamook et al. 2023
Lipase Asahi	*C. viscosum*	Completely degrade PBSA within 4 days.	Arunrattanamook et al. 2023
Lipase F-AP15	*R. oryzae*	Degraded PBSA after cultivation for 22 days.	Arunrattanamook et al. 2023
Lipase-QL	*Alcaligenes* sp.	Degraded PBSA after cultivation for 11 days.	Arunrattanamook et al. 2023
PCL depolymerase	*Brevundimonas* sp. strain MRL-AN1	More than 80% of PCL was degraded within 10 days.	Nawaz et al. 2015
PCL-degrading enzyme	*P. oxalicum* DSYD05	The weight loss can reach 80% after 6 days of cultivation.	Khatua et al. 2024
PCL-degrading enzyme	*C. globosum* ATCC 16,021	The mass loss was as high as 75%, after 28 days.	Vivi et al. 2019
PCL-degrading enzyme	*Sclerotinia* sp. B11IV	The biodegradation activity was 49.7% for PBSA and 33.7% for PCL at 20 °C within 30 days.	Urbanek et al. 2021
PCL-degrading enzyme	*Fusarium* sp. B3’M	The biodegradation activity was 49.7% for PBSA 4.0% and PCL 49.7% at 20 °C within 30 days.	Wang et al. 2019b
Hydrolase RPA1511	*R. palustris*	Degraded almost 40% of PLA within 36 h of incubation.	Wang et al. 2019b

## The degradation process of polymer with additives

**Fig. 5:**
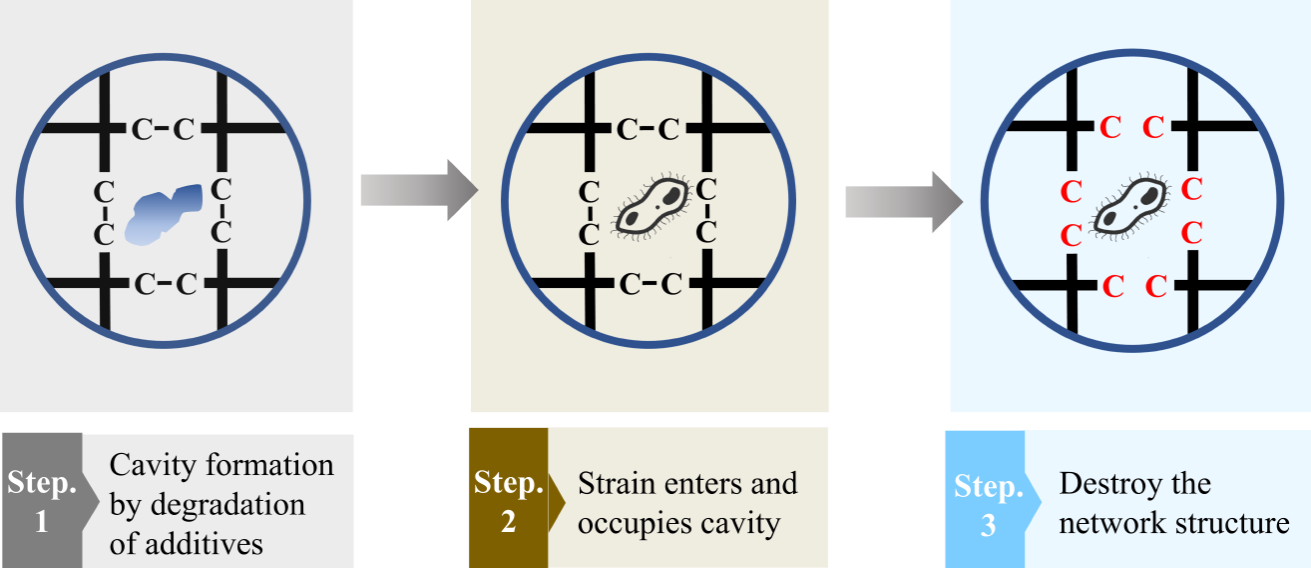
The process of degrading polymers with additives

Additive polymers are a distinct category of polymers whose properties are modified and optimized by the addition of various additives during the manufacturing process, such as emulsifiers, dispersants, and flame retardants. The term used to refer to these substances is “compatibilizers”. For example, flame retardants are added to certain polymers to reduce the flammability of the polymer (Pomata et al. 2024; Vahabi et al. 2021). This method can also enhance the biodegradability of polymers (Bher et al. 2023), the processes for degrading polymers with additives are shown in Fig. 5. Research has demonstrated that employing starch as a compatibilizer enhances the rate of biodegradation and expedites the degradation of PS and PP (Zhang et al. 2022). The composite TPS/MA/PLA is formed by combining polylactic acid with maleic anhydride (MA) and thermoplastic starch (TPS) as compatibilizers. Ricardo observed that pure polylactic acid had a degradation rate of 40.4%, whereas TPS/MA/PLA showed a higher degradation rate of 82% after undergoing a 31-day degradation treatment (Camacho-Muñoz et al. 2020). Furthermore, degradable polymers can be employed as compatibilizers, in conjunction with the incorporation of degradable non-polymers such as starch and maleic anhydride. Finzi et al. included moringa polymers in LDPE and PBAT/PLA blends, resulting in increased mechanical properties and improved biodegradability of the polymers. During the same period, the rate of polymer degradation increased by 80%. Furthermore, the polymer did not break into smaller fragments during degradation, minimizing the potential harm to soil and water (Finzi-Quintão et al. 2019). Olenick et al. conducted degradation studies on a PLA composite with PCL as a compatibilizer. They discovered that the composite disintegrated more rapidly in all of the studied settings (Olewnik-Kruszkowska et al. 2020). Engineered enzymes can also be included in polymers to enhance its degradation. Ting et al. developed a composite structure that consists of an engineered enzyme-protectant polymer, which exposes active areas on the surface of the polymer. This composite structure can be utilized in the production of polyester polymers. Polyesters with this particular structure can completely degrade in a few days (DelRe et al. 2021). The addition of a compatibilizer can accelerate the speed and improve the effectiveness of polymer degradation, however, the Stockholm Convention classifies a number of additives as Persistent Organic Pollutants (POPs) (https://chm.pops.int/Implementation/Alternatives/AlternativestoPOPs/ChemicalslistedinAnnexA/tabid/5837/Default.aspx), such as furans and dioxins, adding these compounds may have a negative impact on the environment and diminish the biodegradability of the polymer. By the way, this advantage is counterbalanced by the increased complexity and expense of production, which can be a barrier to widespread adoption.

## Biodegradation based on multi-strain communities

**Fig. 6:**
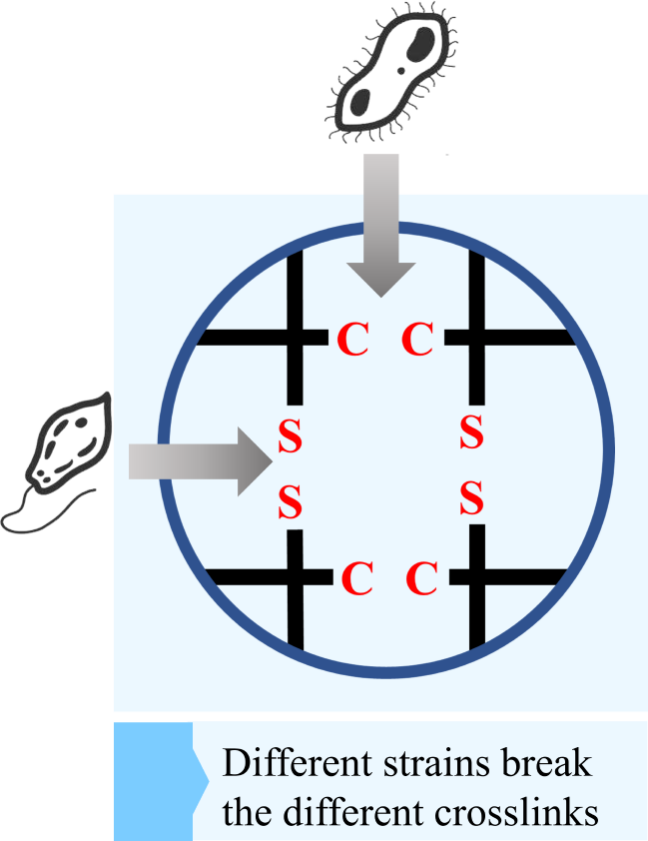
The process of degradation by multiple strains

Previous researches on polymer biodegradation has mostly examined the effects of individual strain on polymer degradation. However, recent studies have demonstrated that multi-strain communities exhibit enhanced efficiency in polymers degradation (Howard et al. 2023), the process of polymer degradation by multiple strains is shown in Fig. 6. In the initial study, Skariyachan et al. discovered that microbial communities had a superior degradation effect compared to single bacteria when cultivating plastic-degrading bacteria using soil and water samples taken from various plastic-contaminated regions in Bangalore, India. They discovered that over a period of 90 days, microbial communities were able to reduce the weight of polymers by 40%, a performance that surpassed individual strains (Ibrahim et al. 2021; Skariyachan et al. 2015). Furthermore, Park’s findings demonstrated that microbial communities have superior degrading capabilities compared to individual strains. They extracted a neutrophilic mixed bacterial community, primarily consisting of *Bacillus* sp. and *Paenibacillus* sp., from the bottom sludge of landfill. After undergoing a 60-day treatment using PE as the sole carbon source, the material experienced a weight reduction of up to 14.7% and a decrease in particle size of 22.8%. These results were notably superior to those achieved by individual strains (Park and Kim 2019). Additionally, a microbial community consisting of multiple actinomycetes is more effective than a single actinomycete at rubber degradation (Nguyen et al. 2020). Recognizing the higher degradation capacities possessed by natural microbial communities, some researchers have attempted to construct artificial microbial communities to facilitate polymer degradation. *Pseudomonas otitidis* strain SPT1, *Bacillus aerius* strain SPT2, *Acanthopleuribacter pedis* strain SPT3, and *Bacillus cereus* strain SPK1 were randomly combined into microbial communities for comparison with single strains on their ability to degrade PVC. It was determined that the combination of microbial communities was more efficient in degrading PVC than single strains (Dhanraj et al. 2022).

Following the discovery that the deterioration caused by microbial communities was more significant than that caused by individual strains, it became imperative to investigate the specific contribution of each strain within the microbial communities. Vagras isolated three microbial communities from El Bordo Poniente landfill, which were named BP1h, BP3h, and BP7h, and deposited at the World Data Centre for Microbiology CFQ100 under the accession numbers CFQ-B-261, CFQ-B-269, and CFQ-B-264, respectively. The researchers examined the capacity of these communities to degrade polyester and polyurethane by measuring the various enzyme activities of the microbiota and assessing the growth potential of particular microbial community strains in these polymers. The majority of the microbial strains in the community were unable to thrive on these polymers independently. Nevertheless, the strains exhibited the ability to thrive on polymers when they were amalgamated to establish a microbial community. The researchers hypothesized that these strains established a network of interactions where each member had a distinct role, enabling them to sustain the growth of the microbial community (Vargas-Suarez et al. 2019). When the strains within a colony establish a stable network, there can be additional advantageous outcomes apart from the enhanced efficiency of the degradation process. Swiontek discovered that a microbial consortium composed of *Aeromonas* and *Rhodococcus* showed a significant ability to degrade polylactic acid (PLA), polyhydroxybutyrate (PHB), and polystyrene (PCL). The increased concentration of bacteriostatic did not impact the degradation ability of the microbial community as a whole. However, the presence of bacteriostatic hindered the degradation ability of an individual strain. These findings suggest that microbial communities exhibit greater resilience to stress compared to individual strains (Swiontek Brzezinska et al. 2020). This implies that microbial communities not only have enhanced degrading capabilities, but also provide a wider range of potential applications due to the presence of several influencing elements in the treatment process. Furthermore, microbial communities exhibit superior stability compared to individual strains. Hence, the exploration of other microbial communities with the capability to degrade polymers is a highly intriguing research endeavor.

## Polymer degradation using enzyme engineering

PET is a polymer formed by ester bonds between terephthalic acid and ethylene glycol. It is widely used, especially in packaging applications such as beverage bottles (Sova et al. 2023; Srivastava et al. 2024). PET has received extensive attention in biodegradation studies. Contemporary molecular biology techniques, including protein engineering and genetic engineering, are primarily applied to enhance the biodegradation of PET.

PET exhibits exceptional durability and retains its structural integrity even after 15 years of natural degradation in outdoor environments, despite belonging to the polyester group (Ioakeimidis et al. 2016). Biodegradation treatments can expedite this process. Microbial production of cutinases is the primary factor responsible for PET biodegradation. These enzymes are quite similar to the other and usually reach their peak activity at the PET glass transition temperature (Tg) of PET, which is approximately 70 °C (Amanna and Rakshit 2023; Richter et al. 2023). *Actinomyces thermophilus* excretes the most typical cutinase (Dąbrowska et al. 2021).However, enzymes from the wild-type strain, are highly susceptible to deactivation near the PET glass transition point and exhibit low thermal stability. Researchers have employed various methodologies to enhance the thermal resistance of enzymes. For instance, research has revealed that the addition of divalent metal ions, such as Ca^2+^ or Mg^2+^ ions, enhances the thermal stability of the hydrolase enzyme, allowing it to degrade PET at a temperature of 65 °C (Qi et al. 2024; Serrano-Aguirre and Prieto 2024). Instead, by substituting metal binding sites with salt bridges or disulfide bonds, the hydrolase may operate at a temperature of 70 °C and degrade PET without the need for divalent metal ions (Zhong-Johnson et al. 2024). Moreover the enzymes under investigation, namely *Fusarium solani pisi* cutinase (FsC), leaf-branch compost cutinase (LCC), and *Thermobifida fusca* hydrolases 1 and 2 (BTA1 and BTA2), have been evaluated for their individual abilities to degrade substances. At a temperature of 65 °C, LCC demonstrated the highest efficiency in degrading PET, with an initial depolymerization rate of 93.2 mgTAeq.h^− 1^mg_enzyme_^−1^ and a 50% degradation rate achieved within 12 h. In addition, the replacement of divalent metal binding sites with disulfide bonds resulted in an improvement in both catalytic activity and thermal stability of the LCC enzyme (Tournier et al. 2020a).The enhancement of enzyme activity through the evolution of enzymes is now possible due to recent advancements in protein and enzyme engineering (Chen et al. 2017; Wang et al. 2017). Similarly, modifications were implemented to cutinase, a pivotal enzyme in the degradation of PET, with the aim of enhancing the efficiency of PET degrade. Meng and Yang developed the mutation design tool Premuse, which they utilized to identify and modify two stable mutants (W159H and F229Y) from a pool of 1486 similar sequences with enhanced enzyme activity. Compared to the wild type, the enzyme’s denaturing temperature and catalytic efficiency (k_cat_/*K*_m_) increased by 10.4 °C and 2 folds, respectively, while its degrading activity surged by nearly 40 folds at 40 °C (Meng et al. 2021). Guo discovered that *Ideonella sakaiensis* 201-F6 has a distinctive cutinase known as IsPETase, which shares a high degree of similarity with LCC in terms of both its sequence and structure, and exhibits a high efficacy in decomposing PET. The hydrolytic activity of the enzyme IsPETase was enhanced by substituting the residues S214 with Ile, and I218 with Ser (Chen et al. 2021). *Bifidobacterium thermophilus* strain TfCut2 produces an IsPETase enzyme that exhibits excellent thermostability. Two enzyme mutants, G62A and G62A/I213s, were created by replacing four conserved amino acids (G62, T63, I178, and I213) of TfCut2 with the corresponding amino acids of LCC. These mutants exhibited enhanced PET hydrolysis activity and improved thermal stability compared to the original LCC enzyme. The hydrolysis activity of G62A increased 4-fold compared to the wild type. In addition, it showed a 5.5-fold decrease in the inhibitor’s binding ability, hence reducing the inhibitor’s impact on the degradation process (Wei et al. 2016).

Determining the enzyme’s crystal structure is crucial for understanding the mechanism of enzyme-catalyzed reactions and providing scientific guidance for enzyme modification. In order to analyze the mechanism of PET degradation catalyzed by IsPET, Austin identified the crystal structure of the IsPET produced by *Ideonella sakaiensi* 201-F6 at a resolution of 0.92 Å, which is the highest-resolution X-ray crystal structure of the apoenzyme available in the database (Austin et al. 2018). Meseguer used the crystal structure resolved by Austin to analyze the cause of the enhanced activity of the PETase mutant (FAST-PETase). By employing classical and hybrid (QM/MM) molecular dynamics (MD) simulations, they determined that the mutation of N233K causes a series of changes that ultimately reduce the catalytic barrier and accelerate the PET degradation reaction (García-Meseguer et al. 2023). Simultaneously with Austin’s IsPET analysis, Fecker analyzed the crystal structure of PETase at a resolution of 2.02 Å. This structure was used in molecular dynamics simulations, giving a firmer theoretical foundation for the evolution of IsPET (Fecker et al. 2018). Tournier utilized molecular docking and enzyme contact-surface analysis strategies to identify mutagenesis sites to improve the catalytic activity of LCC. A total of 209 mutants were obtained by saturation mutagenesis of the screened sites. The most efficient LCC enzyme mutant converted at least 90% of PET to monomer in 10 h, with a degradation efficiency of 16.7 g_hydrolyzed PET_ L^− 1^ h^− 1^ (Tournier et al. 2020b). Despite the achievements, its large-scale applications are still hampered by the remaining 10% of nonbiodegradable PET. Cui et al. designed a mutant of PETase (TurboPETase), with balanced thermostability and hydrolytic capacity, by incorporating a protein language model and force-field-based algorithms. This mutant can nearly completely depolymerize 200 g of PET in 8 h, with a production rate of 61.3 g_hydrolyzed PET_ L^− 1^ h^− 1^ (Cui et al. 2024). Constructing of a large mutant library by computational analysis can greatly accelerate the discovery of enzymes with high heat resistance and high degradation activity. Limited by the low flux of conventional evaluation methods, the amounts of mutant libraries are usually within 10^4^. Cribari proposed a high-throughput yeast surface display platform that can rapidly evaluate mutants with more than 10^7^ enzymes. On this platform, each yeast cell can display different mutants. The enzyme activity is detected by the change of fluorescence during the cleavage of the synthetic probe. Then, the highly active mutants are isolated, which increases the screening flux by 1000 times (Cribari et al. 2023). Previously strategies were based on static protein conformation calculations, which could hardly reflect the dynamic process of catalysis. For this reason, Zheng et al. devised a new computational strategy (affinity analysis based on dynamic docking, ADD) to analyze the ligand affinity energy by molecular docking with the dynamic protein conformations. Compared to static protein conformations, dynamic conformations are more realistic and accurate, which facilitates the discovery of more promising modification sites. The mutant LCC-A2, obtained by the ADD strategy, depolymerized over 90% of PET into terephthalic acid and glycol within 3.3 h at 78 °C. This is currently the fastest PET depolymerization rate on record (Zheng et al. 2024).

Increased enzymatic activity is, essential for PET degradation, as well as for the efficient release of the enzyme into the extracellular environment. Limited studies have been conducted on the synthesis of enzymes that degrade PET. Five distinct Bacillus signal peptides were evaluated for their effect on the secretion of PET hydrolase. The results demonstrated that SP amy generated the highest volume of secretion, almost quadruple that of the native signal peptide SP. In addition, they observed that the upregulation in PET hydrolase expression was triggered by the low-strength P43 promoter. According to the authors’ hypothesis, the weak promoter may allow sufficient time for translation and folding processes. P43 and SP Amy exhibited enhanced degradation activity on PET films (Wang et al. 2020). This suggests that the process of degrading substances is improved by the effective release of enzymes.

PET is the most extensively studied polymer in terms of biodegradation. Currently, researchers are engaged in the development and modification of potent enzymatic agents to significantly enhance the efficiency of PET degradation, rather than identifying efficient microbial strains. Table 4 summarizes the PET degradation capabilities of various PET-degrading enzymes after modification by protein engineering. Although contemporary molecular biology techniques are mainly applied to PET degradation at present, they also open up possibilities for studying other forms of polymer degradation in future advancements.

**Table 4:** Summary of proteins modificated by protein engineering with the ability to degrade PET.

Protein name	Source	Degradation capability	Ref.
Hydrolases 1 and 2	*T. fusca*	Reaching an initial PET-specific depolymerization rate of 3.2 mgTAeq.h^− 1^mg_enzyme_^−1^ at 65 °C.	Tournier et al. 2020a
FsC	*F. solani*	Reaching an initial PET-specific depolymerization rate of 0.01 mgTAeq.h^− 1^mg_enzyme_^−1^ at 65 °C.	Tournier et al. 2020a
LCC	*Leaf-branch compost*	50% PET can be degraded within 12 h, reaching an initial PET-specific depolymerization rate of 93.2 mgTAeq.h^− 1^mg_enzyme_^−1^ at 65 °C.	Tournier et al. 2020a
TfCut2 G62A/I213s	*B. thermophilus* strain KW3	Compared with the wild-type mutant, the degradation activity was increased by 2.7 folds.	Wei et al. 2016
W159H and F229Y	Screen out by Premuse	Compared with the wild type, the degradation activity at 40 °C was nearly 40 folds higher.	Meng et al. 2021
ICCG	LCC enzyme mutant	Degradating at least 90% of PET to monomer in 10 h, with a degradation efficiency of 16.7 g_hydrolyzed PET_ L^− 1^ h^− 1^.	Tournier et al. 2020b
TurboPETase	a mutant of PETase	Depolymerizing 200 g of PET in 8 h, with a production rate of 61.3 g_hydrolyzed PET_ L^− 1^ h^− 1^.	Cui et al. 2024
LCC-A2	obtained by the ADD strategy	Depolymerizing more than 90% of PET into terephthalic acid and glycol within 3.3 h at 78 °C	Zheng et al. 2024

## Conclusions and future perspectives

Due to their exceptional characteristics, polymers are widely employed. However, disposing of waste polymers has always been a challenge because of the lack of appropriate treatment method. Existing methods have their limitations, more precisely, the process of biodegrading polymers is relatively sluggish and biodegradation has a lower resistance to unfavorable settings. Consequently, scientists sought alternate methodologies, such as modifying the enzyme to enhance its ability to degrade substances. However, the majority of bacteria and enzymes lose their activity at elevated temperatures or when exposed to detrimental chemicals such as acids, alkalis, and antibiotics. Hence, additional comprehensive research is necessary before the development of an engineered strain for waste polymer treatment. This involves addressing challenges related to the slow natural degradation process, exploring alternative methodologies, and enhancing the resilience of bacteria and enzymes in various environmental conditions.

Recent research has questioned the conventional method of separating distinct strains for the breakdown of polymers. The benefits of microbial communities have been emphasized. Previous reviews have not specifically highlighted the significance of microbial communities in waste polymer degrading endeavors. This is the inaugural instance where microbial communities have been given equal priority alongside the value of individual strains and synthetic enzymes. Microbial communities have demonstrated superior efficacy in breaking down polymers compared to individual strains. They also exhibit enhanced stability and resistance, particularly when faced with the intricate combinations of polymers commonly found in recyclable garbage. Engineered enzymes have the ability to enhance the speed at which some polymers break down, but they are not yet capable of effectively treating polymers that are combined together. Waste sorting methods are typically rudimentary, generally grouping numerous polymers together in one category. Dealing with intricate polymers using only one specific strain or modified enzyme can provide difficulties. Practical applications derive advantages from the flexibility of multi-strain communities in the treatment of various polymers. Techniques to augment the capacity of a solitary strain to break down polymers can be extended to all strains within the community, while introducing supplementary strains or engineered enzymes can expand the spectrum of polymers that can be decomposed, enhance the efficiency of degradation, and greatly enhance the overall capacity of the community to degrade polymers. In the future, multi-strain degradation of polymers will be the most competitive biodegradation method. Ultimately, biodegradation, whether facilitated by single bacteria, multi-strain communities, or engineered enzymes, now represents the most effective method for managing waste polymers.

While using microbial communities mitigates some limitations associated with single strains, there are emerging issues that need further examination. It is essential to investigate the potential effects of introducing additional strains or enzymes to these communities and assess how pollutants in recycled polymers might impact microbial populations and enzymes. Additional research is required to methodically tackle these obstacles and investigate the potential environmental impact of genetically modified bacteria or enzymes. In summary, despite the unresolved challenges, biodegradation stands out as the most efficient method for dealing with used polymers. Ongoing research and examination of these difficulties will contribute to refining and advancing the process of degrading waste polymers through biodegradation.

### Electronic supplementary material

Below is the link to the electronic supplementary material.

Supplementary Material 1

Supplementary Material 2

## Data Availability

Data will be available on request.

## Abbreviations

PET: polyethylene terephthalate
NR: natural rubber
PE: polyethylene
PP: polypropylene
PVC: polyvinyl chloride
PS: polystyrene
PBS: polybutylene succinate
PBSA: polybutylene succinate-polybutylene adipate
PBAT: polybutylene adipate-butylene terephthalate
PCL: polycaprolactone
PLA: polylactic acid
