# Supplementary figures and images for: Biodegradation: the best solution to the world problem of discarded polymers
Source: Bioresour Bioprocess. 2024 Aug 7;11(1):79. doi: 10.1186/s40643-024-00793-1 (PMC11306678; doi:10.1186/s40643-024-00793-1)

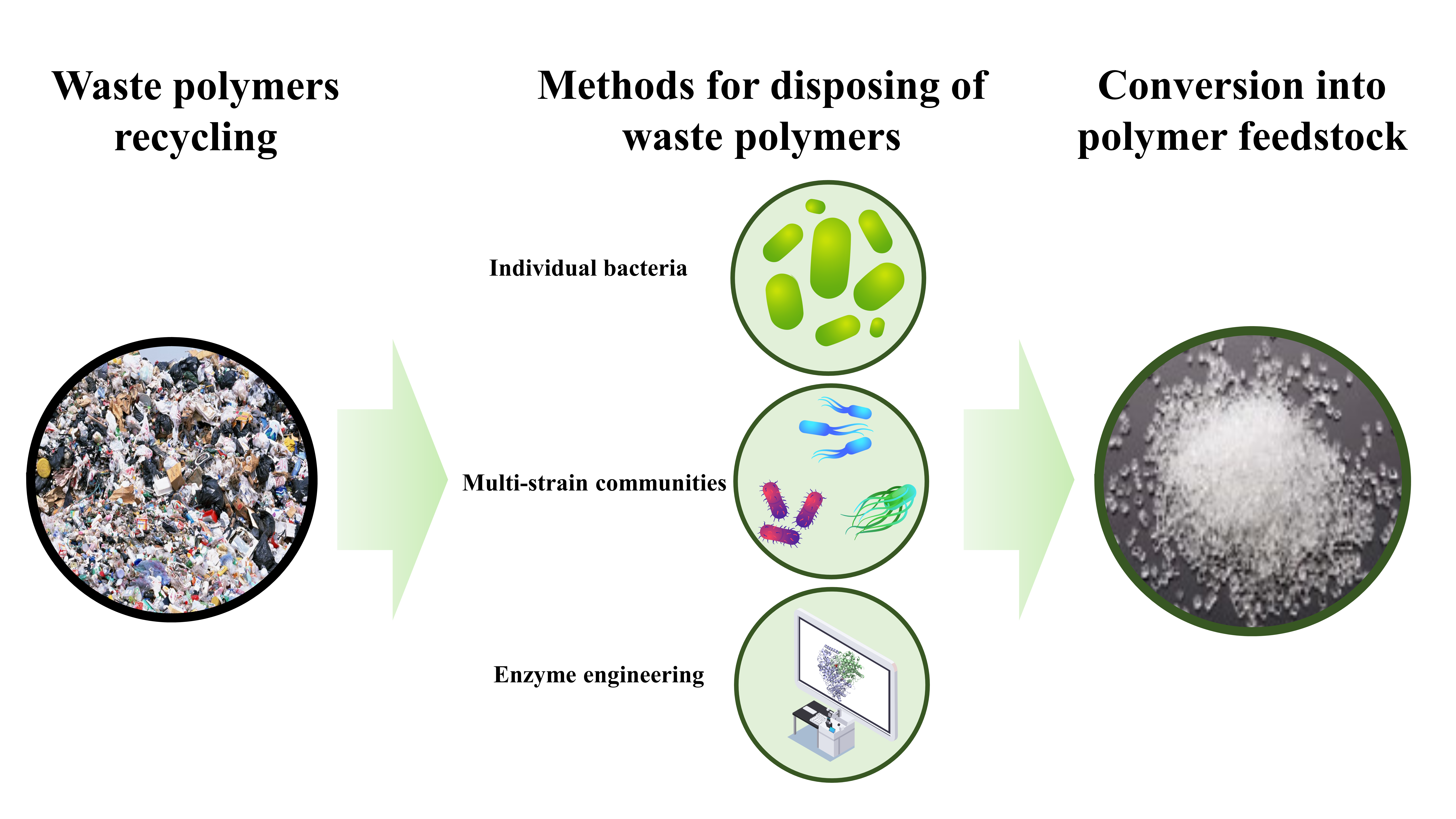

Supplement: Supplementary file 2 — Supplementary Material 2 [file 40643_2024_793_MOESM2_ESM.tif]
