# Supplementary material for: Biodegradation: the best solution to the world problem of discarded polymers
Source: Bioresour Bioprocess. 2024 Aug 7;11(1):79. doi: 10.1186/s40643-024-00793-1 (PMC11306678; doi:10.1186/s40643-024-00793-1)
Supplement: Supplementary file 1 — Supplementary Material 1 [file 40643_2024_793_MOESM1_ESM.docx]

**EIC comments:**

**Response:**

We thank the editor for reviewing our manuscript and providing the professional and constructive comments, which helped us improve the manuscript a lot. We have carefully revised the manuscript and highlighted all the changes in yellow in the revised manuscript.

Learn how to report data properly. Do not propagate bad data reporting. 49.68% is senselessly "accurate." 49.7% is already sufficient (and not that reliable in accuracy without SD). I only listed one example. It is like a disease infecting many inexperienced people.

**Response:**

We appreciate the editor’s comment. We have modified the data in the paper and made adjustment to the majority of them (line 172, line 173, Table 1, line 208, line 289, Table 2, line 321). Only in the case of protein crystal resolution is two decimal places retained, e.g. 0.92 Å (line 446), 2.02 Å (line 451).

"Fig.1" shows poor English. Space obviously missing. Punctuation is literacy too.

**Response:**

Thanks for the editor’s comment. As suggested, we have changed “Fig.1” into “Fig. 1” and modified Fig. 1.

Why did you italicize LCIC4 in Table 1? Senseless to use brackets in Ref. column.

**Response:**

Thanks for the editor’s kind suggestions. We have rectified the incorrect labelling in Table 1 and deleted all brackets from the reference column in Table 1.

Write "Polymers" in the title. It is a countable noun. Improve your English writing in this work.

**Response:**

Thanks for the editor’s comment. As suggested, we revised the title to “Biodegradation: the best solution to the world problem of discarded polymers”. We carefully revised the English writing of the manuscript.

**Reviewer #1:** Comments to the author:

Dear authors, the manuscript presents vital information on the chosen topic and I find it very relevant for the field. Authors are however, required to properly reference and cite the relevant information presented in the manuscript to further strengthen and enhance the scientific quality of the discussion. All typos and omissions should also be checked and corrected throughout the manuscript to enhance readability. Specific comments are given below;

**Response:**

We thank the editor for reviewing our manuscript and providing the professional and constructive comments, which helped us improve the manuscript a lot. We have carefully revised the manuscript and highlighted all the changes in yellow in the revised manuscript.

Title

You may modify as "Biodegradation: the best solution to the world problem of discarded polymer"

**Response:**

Thanks for the reviewer’s helpful comments. The title has been revised to state “Biodegradation: the best solution to the world problem of discarded polymers”.

Abstract

Ok

Keywords: Separate each keyword with semi-colon.

**Response:**

Thanks for your kind suggestions. We use semicolons to separate each keyword.

Introduction

Line 41: comprise not comprised of

**Response:**

Thanks for your kind suggestions. We have changed “comprised of” into “comprise”.

Line 54: delete the initial (W) in the citation

**Response:**

We appreciate the reviewer’s comment. We have deleted the initial (W) in the citation.

Line 59-61: The statement not well constructed. Revise

**Response:**

We appreciate the reviewer’s in-depth comment. To avoid misunderstanding, we have modified the sentence as “The main destinations of the polymers now are shown in Fig. 1.” in line 57.

Figure 1: Needs to be modified and made clearer and easier to understand. What do you mean by USING in the fig. 1?

**Response:**

Thanks for your kind suggestions. We have revised Fig. 1 and added more details to enhance its clarity and improve understanding for readers.

Line 66: Polymer molecular monomers? What is that?

**Response:**

We thank the reviewer comment on this point. We have changed “polymer molecular monomers” into “monomers” in line 63.

Biodegradation based on a single strain

Line 102-111: Provide the appropriate for these facts here. Authors provided information without backing with literature. Revise

**Response:**

Thanks for your kind suggestions. We have cited recent scholarly references and added relevant evidence to substantiate this part using study in page 6, line 98-108. “Biodegradation reactions vary among different polymers; for example, Pseudomonas citronellolis was able to degrade PVC but not PP (Giacomucci et al. 2019). Therefore, we categorize biodegradation based on the specific polymer type, such as rubber, plastics, and polyester. Rubber is a naturally occurring polymer composed of cis-1,4-polyisoprene (Andler 2020). However, natural rubber has lower stability and abrasion resistance. Typically, natural rubber undergoes a process called vulcanization to create vulcanized rubber (Wiśniewska et al. 2022). This process leads to rubber with enhanced properties, including greater stability, abrasion resistance, and lower degradability. Plastics are synthetic materials created by combining or condensing monomers, such as polyethylene (PE), polypropylene (PP), polyvinyl chloride (PVC), and polystyrene (PS), which are widely utilized. The primary distinction between plastics and rubber lies in their deformation behavior. Plastic deforms plastically, while rubber deforms elastically (Zhigang and Kuangdi 2022).”

Figure 3: The description of the steps in the figure should be revised. For instance, in step 1, authors can see "strain enters the network structure" not just "Enter the network structure" The main components of the figure should well-labelled for better understanding.

**Response:**

Thanks for your kind suggestions. We have changed “Enter the network structure” into “Strain enters the network structure” and revised the explanations of the individual stages and redesigned the diagram to enhance its brevity in the modified Fig. 3.

Line 128: Merge the citations into a single parenthesis.

**Response:**

We appreciate the reviewer’s comment. We have combined the citations (Page 8, line 128).

Line 133: easy to distinguish not easily to distinguish

**Response:**

We appreciate the reviewer’s comment. We have replaced the “easily to distinguish” by “easy to distinguish” in page 8, line 135.

Line 147: Use lower case for gram-negative.

**Response:**

As suggested by the reviewer, we have changed “Gram-negative” into “gram-negative” in page 9, line 149 and 154.

Line 169-170: Italicise the scientific names

**Response:**

Thanks for your kind suggestions. We have italicized the scientific names (Page 10, line 171-172).

Degradation of the plastic family

Line 230: check spelling of properties

**Response:**

As suggested by the reviewer, the wrong word was corrected as “properties” in page 13, line 232.

Italicise B. cereus in Table 2

**Response:**

We appreciate the reviewer’s comment. We have italicized the scientific names in page 15, Table 2.

Degradation of the polyester family

Line 267: Italicise scientific name

**Response:**

Thanks for your kind suggestions. We have italicized the scientific names (Page 16, line 268).

The degradation process of polymer with additives

Line 314: Optimized by. Note "by" is omitted.

**Response:**

Thanks for the reviewer’s comment. We have changed “optimized” into “optimized by” (Page 19, line 316).

Figure 4: The figure can made better by further description and labels.

**Response:**

As suggested, we substitute the phrases "Degradate the additives" and "Occupy the cavity" with "Cavity formation by degradation of additives" and "Strain enters and occupies cavity" in the modified Fig. 4.

Biodegradation based on multi-strain communities

Line 472: Delete "of"

**Response:**

We thank the reviewer comment on this point. We have changed “upregulation of” into “upregulation” (Page 28, line 480).

Conclusions and Perspectives

Line 489: Replace "Regrettably" with a better word.

**Response:**

We appreciate the reviewer’s comment. And we have made revision to this section. “However, the majority of bacteria and enzymes lose their activity at elevated temperatures or when exposed to detrimental chemicals such as acids, alkalis, and antibiotics.” (Page 29, line 498-500).

Line 490: which impeding? Please properly write

**Response:**

Thanks for your kind suggestions. Challenges such as extended duration, inadequate durability, and limited scope of use impede the widespread biodegradation of polymers. The sentence was corrected as “Existing methods have their limitations, more precisely, the process of biodegrading polymers is relatively sluggish and biodegradation has a lower resistance to unfavorable settings.” (Page 29, line 496-497).

Line 493-496: This should be moved to the main content of the manuscript since it is not appropriate for this section. This section should mainly focus on highlighting the main points discussed in the main content and also present future perspectives. It is not a section to bring more literature.

**Response:**

We appreciate the reviewer’s comment. As suggested, we eliminated the citation and rephrased the passage. The sentence was rewritten as “Consequently, scientists sought alternate methodologies, such as modifying the enzyme to enhance its ability to degrade substances. However, the majority of bacteria and enzymes lose their activity at elevated temperatures or when exposed to detrimental chemicals such as acids, alkalis, and antibiotics. Hence, additional comprehensive research is necessary before the development of an engineered strain for waste polymer treatment. This involves addressing challenges related to the slow natural degradation process, exploring alternative methodologies, and enhancing the resilience of bacteria and enzymes in various environmental conditions.” (Page 29, line 497-500).

Line 527: X. Shen contributed the manuscript? Please properly present this.

**Response:**

Thanks for your kind comment. We have changed “J. Wu., X. Shen contributed the manuscript.” into “J. Wu and X. Shen wrote and revised the manuscript.” in page 31, line 542.

References

All scientific names should be italicised throughout the reference list

**Response:**

As suggested by the reviewer, we have italicized the scientific names (line 563, 577, 618, 624, 639-641, 657-658, 663, 690, 935, 737, 750, 753, 759, 780, 787, 790, 800).

Figure 3: Revise as mentioned previous

**Response:**

Thanks for your kind suggestions. We have revised it as requested earlier.

Figure 4: More description required for clarity

**Response:**

We thank the reviewer comment on this point. As suggested, we substitute the phrases “Degradate the additives” and “Occupy the cavity” with “Cavity formation by degradation of additives” and “Strain enters and occupies cavity” in the modified Fig. 4.

Figure 1: Modify the figure for clarity purposes. What do you mean by USING?

**Response:**

We thank the reviewer for the constructive comments. As suggested, we revised Fig. 1 by incorporating more details to enhance reader comprehension.

Figure 2: Repetition of the figure

**Response:**

Thanks for the reviewer’s comment. As suggested, we have revised the explanations of the individual stages and redesigned the diagram to enhance its brevity.

Graphical Abstract: Should be improved for clearer understanding and readability.

**Response:**

We thank the reviewer for the constructive comments. We have included the requisite clarifying annotations to facilitate comprehension.

**Reviewer #2**: Jun Wu et al., described in this review paper about summary of polymer biodegradation from literatures. Authors summarized about discarded polymers such as rubber, plastics, polyesters, and so on, and described current situation of biodegradation by microbes. Basically, authors summarized clearly about current situation, and well-described.

**Response:**

We thank the reviewers for reviewing our manuscript and providing the professional and constructive comments, which helped us improve the manuscript a lot. We have carefully revised the manuscript and highlighted all the changes in yellow in the revised manuscript.

However, there are not clearly mentioned about uniqueness of this review paper compared with other review paper related in polymer biodegradation. Authors need to justify why this review paper is necessary to publish as review, and explain clearly about uniqueness of this current review paper compared with others. Otherwise, reader might not see any values to read this review paper.

**Response:**

Thanks for the reviewer’s helpful comments. Previous reviews have largely focused on single bacteria and engineered enzymes for polymer degradation, often undervaluing the role of multi-strain communities. Our review is poised to be the first to exclusively explore the role of multi-strain communities in polymer degradation. Our research indicates that multi-strain communities offer significant advantages over single bacteria, particularly in terms of durability and versatility across a broader range of polymer types. As suggested, we emphasized this perspective in the conclusions and future perspectives section. Thus, publications like ours are desperately needed to support and highlight the benefits of multi-strain deteriorated polymers so that more readers and researchers will be aware of this trend and the field will advance. As a result, we think that releasing this piece is essential and that our review is irreplaceably unique.

Moreover, it'll be suggested that authors could summarize more about additives of polymer, and the effect of additives for the biodegradation. For example, some of polymer containing flame retardants to provide less flammable to selective polymer, and percentages of flame retardant could be around 1 to 10% (https://www.sciencedirect.com/science/article/pii/S0927796X20300620). Also, some of additives could be categorized as POPs (https://chm.pops.int/Implementation/Alternatives/AlternativestoPOPs/ChemicalslistedinAnnexA/tabid/5837/Default.aspx). If authors could summarize about the effect of additives of biodegradation, not only for the main components of polymers, and this could be good motivation to read it as unique point of this review paper. Overall, it would be suggested that current version of review paper might be not acceptable for the publication in Bioresources and Bioprocessing because of lacking uniqueness and justification of current summary compared with other literatures.

**Response:**

We appreciate the reviewer’s comment. We added discussion of additional substances such as dispersants, emulsifiers, and flame retardants, and present instances illustrating the impact of these substances on the characteristics of polymers in the lines 315-319.

“Additive polymers are a distinct category of polymers whose properties are modified and optimized by the addition of various additives during the manufacturing process, such as emulsifiers, dispersants, and flame retardants. The term used to refer to these substances is "compatibilizers". For example, flame retardants are added to certain polymers to reduce the flammability of the polymer (Pomata et al. 2024; Vahabi et al. 2021).”

The effect of most additives on the degradability of polymers is uncertain, except for those compounds that enhance polymer degradation. The text provides examples of such typical compounds.

Of course, certain additional compounds also decrease degradability; in lines 338–345, we address the possible impacts of these additives on the biodegradability of polymers as well as the potential environmental risks associated with the release of additives following polymer decomposition.

“The addition of a compatibilizer can accelerate the speed and improve the effectiveness of polymer degradation, however, the Stockholm Convention classifies a number of additives as Persistent Organic Pollutants (POPs) (https://chm.pops.int/Implementation/Alternatives/AlternativestoPOPs/ChemicalslistedinAnnexA/tabid/5837/Default.aspx), such as furans and dioxins, adding these compounds may have a negative impact on the environment and diminish the biodegradability of the polymer. By the way, this advantage is counterbalanced by the increased complexity and expense of production, which can be a barrier to widespread adoption.”

**Reviewer #3**:

**Response:**

We thank the reviewers for reviewing our manuscript and providing the professional and constructive comments, which helped us improve the manuscript a lot. We have carefully revised the manuscript and highlighted all the changes in yellow in the revised manuscript.

Title

1- Modify your title to make more suitable to your manuscript

2- It seems a sentence in your text

3- You could say Biodegradation is the best ….etc or make it as a question, Is biodegradation …..etc.

**Response:**

Thanks for your kind suggestions. “Biodegradation: the best solution to the world problem of discarded polymers” replaced the original title “The best solution to the world problem of discarded polymer: biodegradation”.

Abstract

1- Improved our quality of life (explain how briefly).

**Response:**

We thank the reviewer for the constructive comments. As suggested, we revised this sentence “The widespread use of polymers has made our lives increasingly convenient by offering a more convenient and dependable material.” in page 2, line 22-23.

2- Use "biodegradation" term wherever you refer to a biological possess of degradation do not use "degradation"

**Response:**

Thanks for the reviewer’s comment. As suggested, we have changed “degradation” into “biodegradation” in page 2, line 26, 28-29.

3- In one sentence mention your main conclusion in the abstract.

**Response:**

As suggested by the reviewer, we added following sentences in manuscript to comment on this point. “Ultimately, biodegradation, whether facilitated by single bacteria, multi-strain communities, or engineered enzymes-remains, now represents the most effective method for managing waste polymers.” in page 2, line 31-33.

4- Rewrite the last paragraph.

**Response:**

We thank the reviewer for the constructive comments. As suggested, the paragraph was corrected as “In this review, we assess the current state of these three significant fields of research, provide practical solutions to issues with polymer biodegradation, and outline potential future directions for the subject. Ultimately, biodegradation, whether facilitated by single bacteria, multi-strain communities, or engineered enzymes-remains, now represents the most effective method for managing waste polymers.” in page 2, line 29-33.

5- The output of your graphical abstract is not clear, what is that? Explain the figure?

**Response:**

We thank the reviewer comment on this point. For better comprehension, we have added stage descriptions, added “Waste polymers recycling” “Methods for disposing of waste polymers” and “Conversion into polymer feedstock” as descriptive statements.

6- Avoid using two words in the keywords section, try to use one word.

**Response:**

As suggested, we have changed “polymer degradation” into “polymer” (Page 3, line 38). However, it is indeed accurate that certain words prove challenging to substitute with a single word. We appreciate the reviewer’s comment on this issue and we hope the reviewer can understand that situation and agree with our decision.

Introduction

1- Be consistence use tons or metric tones

**Response:**

Thanks for your kind suggestions. “tones” was corrected as “tons”.

2- Why did you put your conclusion in the middle of the introduction?

**Response:**

Thanks for the reviewer’s comment. Our conclusion is “In the future, multi-strain degradation of polymers will be the most competitive biodegradation method. Ultimately, biodegradation, whether facilitated by single bacteria, multi-strain communities, or engineered enzymes, now represents the most effective method for managing waste polymers.” (Page 30, line 523-526) The statement is positioned in the center of the text to highlight the benefits of biodegradation compared to landfill incineration, “Biodegradation is the most appropriate method” is a more general perception and has been stated in several articles. Such as:

1. Kim, M. S., Chang, H., Zheng, L., Yan, Q., Pfleger, B. F., & Klier, J., et al. (2023). A review of biodegradable plastics: chemistry, applications, properties, and future research needs. Chem. Rev. 123 （16）： 9915–9939 <https://doi.org/10.1021/acs.chemrev.2c00876>
2. Lambert S, Wagner M (2017) Environmental performance of bio-based and biodegradable plastics: The road ahead. Chem Soc Rev 46 (22):6855-6871 https://doi.org/10.1039/c7cs00149e
3. Samir, A., Ashour, F.H., Hakim, A.A.A. et al. (2022). Recent advances in biodegradable polymers for sustainable applications. npj Mater Degrad 6, 68 <https://doi.org/10.1038/s41529-022-00277-7>
4. Filiciotto L, Rothenberg G (2021) Biodegradable plastics: Standards, policies, and impacts. ChemSusChem 14 (1):56-72 https://doi.org/10.1002/cssc.202002044
3- Rewrite the paragraph starts line 68 ends line 73.

**Response:**

Thanks for your kind suggestions. As suggested by the reviewer, the paragraph was corrected as “Biodegradation, when contrasted with traditional landfill methods, has the ability to preserve valuable land resources by reducing solid volume by up to 80% (Mostafa et al. 2018; Rai et al. 2021). Moreover, biodegradation exhibits a superior capacity for degradation. The rapid decomposition of the polyester polymer polyethylene terephthalate (PET) exemplifies the high efficiency of biodegradation. Contrary to the lengthy natural deterioration process that takes place in landfills over several decades, biodegradation can rapidly disintegrate PET within a few days.” in page 4, lines 63-69.

4- What do you mean by "recycling of plastic by microorganisms"?

**Response:**

Thanks for the reviewer’s constructive comment. We have rewritten it as “Biodegradation of waste polymers with the involvement of microbes” in Fig. 2.

5- Figure 2 is confusing please rearrange it.

**Response:**

We appreciate the reviewer’s comment and rearrange it.

6- Do you think genetically modified bacteria or enzymes are save to the environment?

**Response:**

Thanks for the reviewer’s comment. We believe that greater research and discussion are necessary before any conclusions can be drawn on the issue of whether genetically engineered bacteria or enzymes might be harmful to the environment, as indicated in lines 522–524.

Biodegradation based on a single strain

1- Figure 3 this is not a process of degradation, please fix it in the text as well

**Response:**

We thank the reviewer for the constructive comments. As suggested, we fix it in the text in page 7, line 112-114. We have made revision to Fig. 3 and the descriptions of the different stages. Additionally, we have redone the figures to enhance their conciseness.

2- Examples of single strain degraders need to be mentioned here.

**Response:**

Thanks for your kind suggestions. We have included references to scholarly works in order to provide empirical evidence for the content of this section in page 6, line 99-100. “Biodegradation reactions vary among different polymers; for example, *Pseudomonas citronellolis* was able to degrade PVC but not PP (Giacomucci et al. 2019).”

The degradation process of rubber

1- Rewrite the paragraph starts line 122 ends line 125.

**Response:**

We thank the reviewer comment on this point. The paragraph was corrected as “The original researchers investigated how well a variety of wild bacteria might degrade rubber using randomized trials. *Rhodococcus pyridinivorans* F5 exhibited the highest efficiency in degrading natural rubber, resulting in an 18% decrease in rubber weight over a 30 days period.” in page 8, line 124-127.

2- Fix the references order (line 128).

**Response:**

We appreciate the reviewer’s comment. We have combined the citations (Page 8, line 128).

3- What do you mean by translucent halo? Maybe you need to explain the prosses and which step is this?

**Response:**

We thank the reviewer for the constructive comments. As suggested, we added descriptions “They could produce hyphae and create transparent areas when decomposing rubber on agar plates with NR.” in lines 132 to 133.

4- Font has to be italic for all genus and species names (lines 169-170) fix it in all the manuscript

**Response:**

Thanks for your kind suggestions. As suggested, we have carefully italicized all the scientific names in our manuscript.

Degradation of the plastic family

1- I think in review articles it is a good idea to put the chemical structures for the compounds that you are talking about such as PE, PP PCV etc.….

**Response:**

We thank the reviewer comment on this point. We agree with the reviewers that it is a good idea to include the chemical structure of the compounds discussed in the text. As suggested, we have included Fig. 4 and provided a detailed description of it in the main text to enhance the visualization of the compounds' structure. “The process of polymer degradation by a single strain is shown in Fig. 3 and Fig. 4 depicts the chemical structures of the majority of the polymers mentioned in the text.”

2- Reorganize table2

**Response:**

We thank the reviewer for the constructive comments. As suggested, we have realigned Table 2.

3- Again, I did not see the biodegradation mechanism

.

**Response:**

Thanks for the reviewer’s comment. We have made revision to Fig. 5 and the descriptions of the different stages. Our diagram aims to illustrate the process via which degradation takes place, rather than the mechanism of deterioration.

Degradation of the polyester family

1- Yes, this is a good part

**Response:**

We thank the reviewer comment on this point.

The degradation process of polymer with additives

1- Do you think the additives effect the quality of polymers or just enhance the biodegradability?

**Response:**

Thanks for the reviewer’s comment. Additives have a significant impact on the quality of polymers in various aspects, including flame retardancy, stability, and not limited to enhancing biodegradability. The inclusion of additives to augment the degradability of polymers is just one outcome. Our primary focus is on the degradation of polymers, thus the majority of the section enumerates compounds that boost degradability.

2- Again, the figure is confusing.

**Response:**

We thank the reviewer for the constructive comments. As suggested, we have recreated the graphics and changed the descriptions.

Biodegradation based on multi-strain communities

1- Do you think using multi-strains always better in biodegradation?

**Response:**

Thanks for the reviewer’s comment. In real application circumstances, the utilization of numerous strains is consistently more advantageous. As outlined in the research, employing multiple strains can enhance resistance to challenging environments and enable simultaneous degradation of various polymers. The similarly described is shown in page 29, line 518-522.

2- What are these abbreviations (BP1h, BP3h, and BP7h)? explain in the text please

**Response:**

We appreciate the reviewer’s comment. To clarify this detail, we have rewritten it as “Vagras isolated three microbial communities from El Bordo Poniente landfill, which were named BP1h, BP3h, and BP7h, and deposited at the World Data Centre for Microbiology CFQ100 under the accession numbers CFQ-B-261, CFQ-B-269, and CFQ-B-264, respectively.” in page 22, line 375-378.

Polymer degradation using enzyme engineering

1- Why you focused on enzymes ability function at high temperature?

**Response:**

Thanks for your kind suggestions. Enzymes that break down PET are most effective at 70 °C, therefore, most research has concentrated on enhancing the thermal stability to ensure optimal degradation.

2- You did not mention which one of three ways is better to use in biodegradation?

**Response:**

We appreciate the reviewer’s constructive comment. As suggested, we have modified the discussion “Recent research has questioned the conventional method of separating distinct strains for the breakdown of polymers. The benefits of microbial communities have been emphasized. Previous reviews have not specifically highlighted the significance of microbial communities in waste polymer degrading endeavors. This is the inaugural instance where microbial communities have been given equal priority alongside the value of individual strains and synthetic enzymes. Microbial communities have demonstrated superior efficacy in breaking down polymers compared to individual strains. They also exhibit enhanced stability and resistance, particularly when faced with the intricate combinations of polymers commonly found in recyclable garbage. Engineered enzymes have the ability to enhance the speed at which some polymers break down, but they are not yet capable of effectively treating polymers that are combined together. Waste sorting methods are typically rudimentary, generally grouping numerous polymers together in one category. Dealing with intricate polymers using only one specific strain or modified enzyme can provide difficulties. Practical applications derive advantages from the flexibility of multi-strain communities in the treatment of various polymers. Techniques to augment the capacity of a solitary strain to break down polymers can be extended to all strains within the community, while introducing supplementary strains or engineered enzymes can expand the spectrum of polymers that can be decomposed, enhance the efficiency of degradation, and greatly enhance the overall capacity of the community to degrade polymers. In the future, multi-strain degradation of polymers will be the most competitive biodegradation method. Ultimately, biodegradation, whether facilitated by single bacteria, multi-strain communities, or engineered enzymes, now represents the most effective method for managing waste polymers.” in page 29, line 506-526.

Conclusions

1- You need to make it shorter focus on the importance of this work.

**Response:**

Thanks for the reviewer’s comment. As suggested, we have condensed the content to emphasize the significance of this project.

References

1- Double check your references and fix some of them please.

**Response:**

Thanks for your kind suggestions. We conducted a thorough review of the references and made necessary corrections, and properly italicizing the scientific name of the strain (line 563, 577, 618, 624, 639-641, 657-658, 663, 690, 935, 737, 750, 753, 759, 780, 787, 790, 800).
